# Target decoupling in coupled systems resistant to random perturbation

**DOI:** 10.1038/s41598-017-01241-1

**Published:** 2017-05-19

**Authors:** Sunkyu Yu, Xianji Piao, Namkyoo Park

**Affiliations:** Photonic Systems Laboratory, Department of Electrical and Computer Engineering, Seoul National University, Seoul, 08826 Korea

## Abstract

To suppress unwanted crosstalks between nearby optical elements, the decoupling technique for integrated systems has been desired for the target control of light flows. Although cloaking methods have enabled complete decoupling of optical elements by manipulating electromagnetic waves microscopically, it is difficult to be applied rigorously to control each unit element in coupled systems due to severe restrictions on material parameters for cloaking. Here we develop the macroscopic approach to design crosstalk-free regions in coupled optical systems. By inversely designing the eigenstate which encompasses target elements, the stable decoupling of the elements from the coupled system is achieved, being completely independent from the random alteration of the decoupled region, and at the same time, allowing coherent and scattering-free wave transport with desired spatial profiles. We also demonstrate the decoupling in disordered systems, overcoming the transport blockade from Anderson localization. Our results provide an attractive solution for “target hiding” of elements inside coupled systems.

## Introduction

Invisibility cloaking is one of the most fascinating achievements in transformation optics^1–3^. The coordinate transformation between virtual and physical spaces provides the rigorous design guidance of material parameters, perfectly separating the light flow in the cloaked region from that in the other part. Although transformation optics derived from full-vectorial Maxwell’s equations^1^ successfully provides an exact solution for omnidirectional and scattering-free perfect cloaking, at the same time, its strict demand on material designs has caused hardship to the practical implementation of the cloaking in spite of recent achievements in optical metamaterials^4^.

The stringent condition of rigorous transformation optics has also hindered the application of the cloaking to photonic integrated circuits which require the “decoupling” technique^5, 6^ between elements for crosstalk-free signal transport. Consider the ‘hiding’ (or ‘decoupling’) of some elements inside densely packed coupled optical systems^5, 7–11^. Transformation optics in this scenario provides the severely intricate solution even for the approximated case^12^: the coating of target elements with spatially-varying, highly anisotropic metamaterials of extreme material parameters (effective permittivity ~0), which derives the ‘microscopic’ removal of the coupling to the target elements. We note that similar restrictions can also be found in other alternative cloaking methodologies. The cloaking using accidental degeneracy^13^ requires the well-defined crystalline structure to maintain the Dirac point, and thus cloaked elements should be separated by more than several lattice periods, prohibiting the integration. Although the concept of parity-time symmetry has been applied to the unidirectional invisibility in one-dimensional coupled structures^14, 15^ based on their singular scattering, the extension to multi-dimensional integrated systems encounters the similar difficulty with transformation optics: the coating of spatially varying gain-loss media^16^ for each element. The optical analogy of the adiabatic passage^5, 17^ has also been employed to hide the inner waveguide in tri-atomic designs, but its multi-dimensional or *N*-atomic realization still remains as a challenge.

Here, we propose the ‘macroscopic’ approach to the decoupling based on the eigenstate molding applicable to *N*-atomic coupled optical systems, instead of the microscopic material arrangement for each element^1–3, 13, 16^. We demonstrate that the scattering-free perfect transmission can be achieved through the system eigenstate which includes target decoupled elements, against the random perturbation of the self-energy inside the target region of the system. By controlling the self-energy of the system in a moderate range, the designer spatial profile of the wave flow can also be achieved around target elements, while preserving the scattering-free condition. Utilizing the generality of our eigenstate decoupling method, we also show the stable decoupling in disordered systems for the first time, which resolves the blockade of wave transport from Anderson localizations^18, 19^.

## Results

### Concept of target decoupling

**Figure 1:**
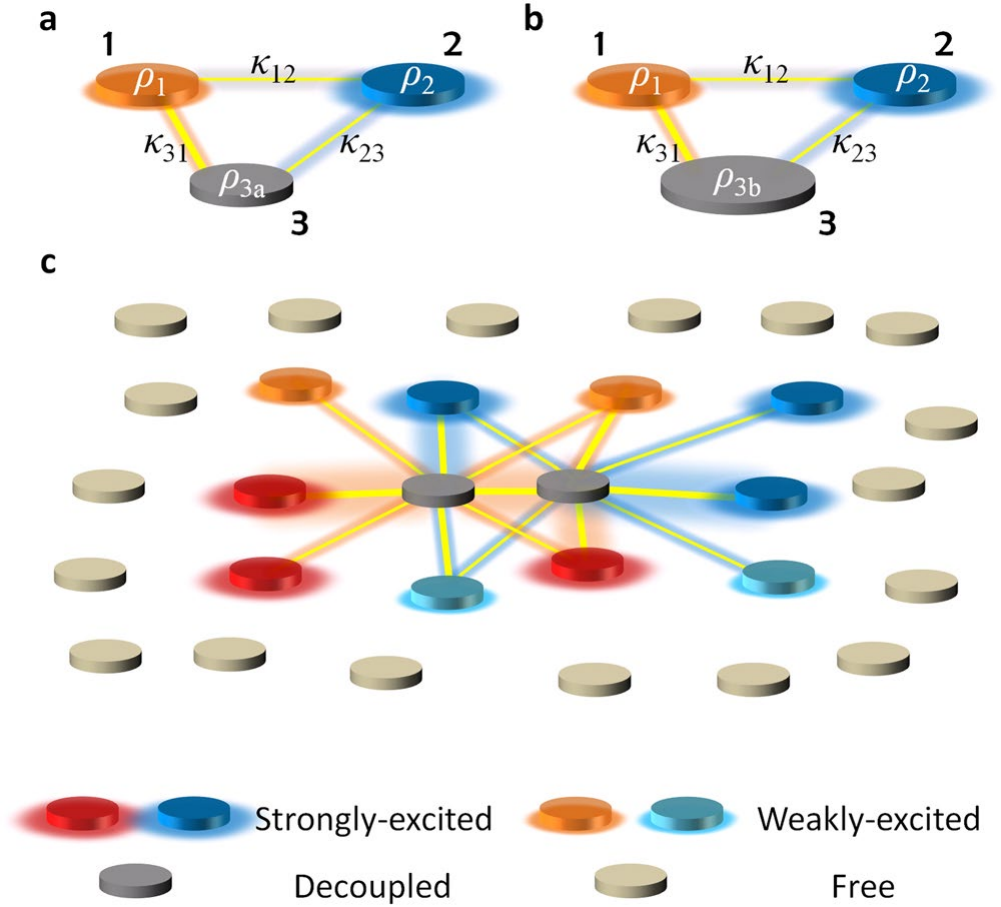
Schematics of the designer state for the decoupling in coupled optical systems. Tri-atomic examples for different self-energy at the 3^rd^ element: (**a**) *ρ*_3a_ and (**b**) *ρ*_3b_. *ρ* for self-energy of each element and *κ* for the coupling between elements in (**a,b**). **(c)** *N*-atomic example for two target decoupled elements at the center (dark gray). Coupling is denoted as the line between elements, and for clarity, coupling terms only around the target elements are presented.

We begin with an instructive example of a triatomic system where each element has the self-energy of *ρ*_*i*_ (*e.g*. resonant frequency *f* of an uncoupled resonator), and the coupling between the *i*-th and *j*-th elements is given as *κ*_*ij*_ (Fig. 1a, *κ*_*ij*_ ~ *κ*_*ji*_ for the similar shape of elements^20^). The system then satisfies the following Hamiltonian equation^5, 10, 21^

1 $$[\begin{array}{ccc}{\rho }_{1} & {\kappa }_{12} & {\kappa }_{13}\\ {\kappa }_{21} & {\rho }_{2} & {\kappa }_{23}\\ {\kappa }_{31} & {\kappa }_{32} & {\rho }_{3}\end{array}]\,[\begin{array}{c}{\psi }_{1}\\ {\psi }_{2}\\ {\psi }_{3}\end{array}]=\rho [\begin{array}{c}{\psi }_{1}\\ {\psi }_{2}\\ {\psi }_{3}\end{array}],$$

for the field amplitude at each element Ψ = [*ψ*_1_, *ψ*_2_, *ψ*_3_]^T^. We establish the decoupling of the 3^rd^ element, calling for the invariant eigenstate for the random perturbation of *ρ*_3_ (Fig. 1a *versus* 1b, as *ρ*_3a_ ≠ *ρ*_3b_). From the setting of *ψ*_3_ = 0 to remove the *ρ*_3_-dependency, *i.e*. ‘hiding’ of the 3^rd^ element in the target eigenstate, Eq. (1) then derives the condition of *κ*_31_·*ψ*_1_ + *κ*_32_·*ψ*_2_ = 0 which corresponds to the destructive coupling interference in the 3^rd^ element (Fig. 1a,b). This condition applied to Eq. (1) defines the necessary condition of the self-energy for decoupling the 3^rd^ element as *ρ*_1_ − *ρ*_2_ = *κ*_31_·*κ*_12_/*κ*_32_ − *κ*_32_·*κ*_21_/*κ*_31_, and the corresponding eigenvalue of the target eigenstate can be controlled by *ρ* = *ρ*_1_ − *κ*_31_·*κ*_12_/*κ*_32_. Hence, by controlling the self-energy of the elements (*ρ*_1,2_) which have the given coupling network (fixed *κ*_*ij*_), we can “hide” some elements inside the coupled system at the desired eigenvalue *ρ*, for any networks even including irregular or symmetry-broken cases (e.g. *κ*_23_ ≠ *κ*_31_). We note that this approach can be easily extended to hiding *m*-elements inside *N*-atomic systems (Fig. 1c, Supplementary Note 1). Interestingly, although the nearby elements (blue and red elements in Fig. 1c) of the target region (2 dark gray elements in the center, Fig. 1c) should have the designed field distribution for the decoupling, the field at the rest elements (light gray elements in Fig. 1c) of the system can be controlled irrespective of the decoupling (Supplementary Note 1 and Fig. S1c,d), allowing the scattering-free designer wave flow around the decoupled region.

### Target decoupling in coupled optical systems

**Figure 2:**
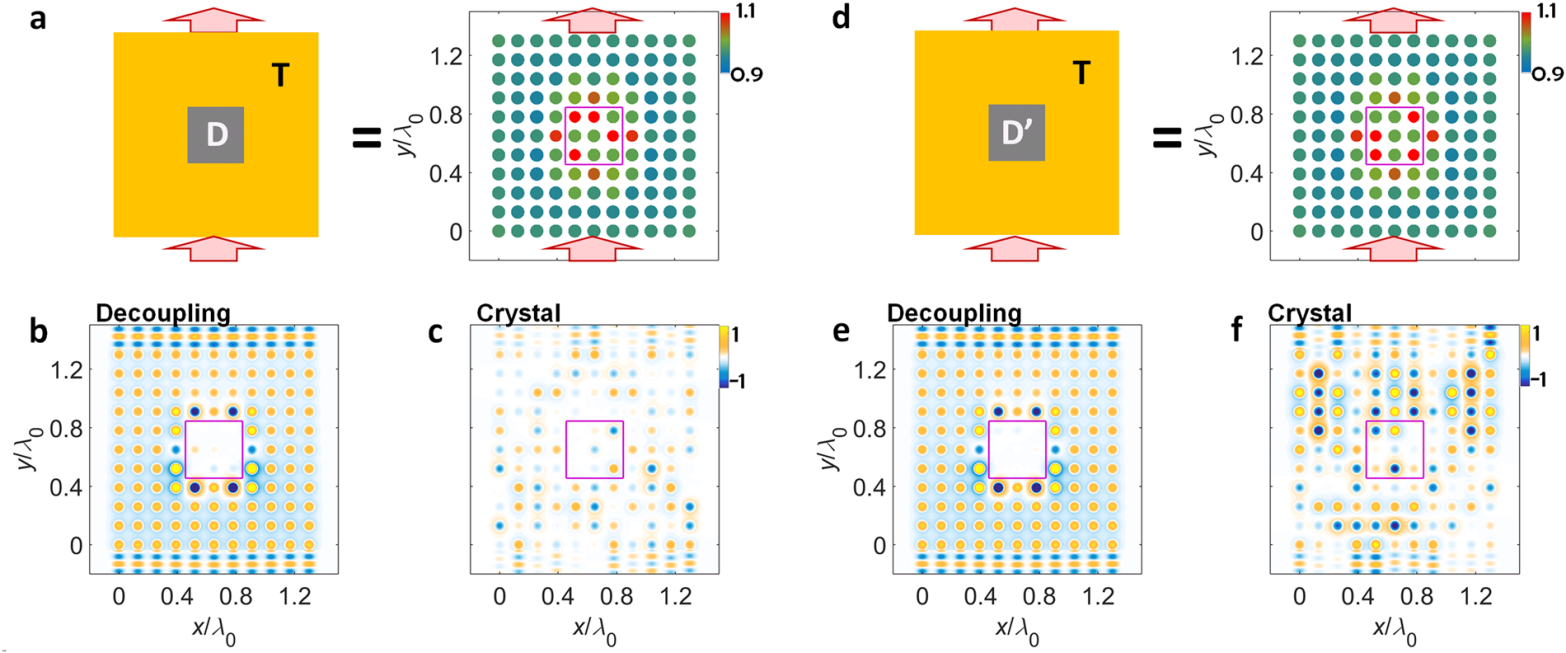
Demonstration of eigenstate decoupling for planewave input and output waves through the crystal lattice. The different configurations in the decoupled region are compared for the cases of (**a–c**) and (**d–f**) (D ≠ D’, red boxes in the right panels of (**a,d**)). The decoupling results in (**b,e**) are compared with the results of ordinary crystal systems in (**c,f**) composed of identical elements. *λ*_0_ is the free-space wavelength, and all of the design parameters are shown in Supplementary Note 2.

**Figure 3:**
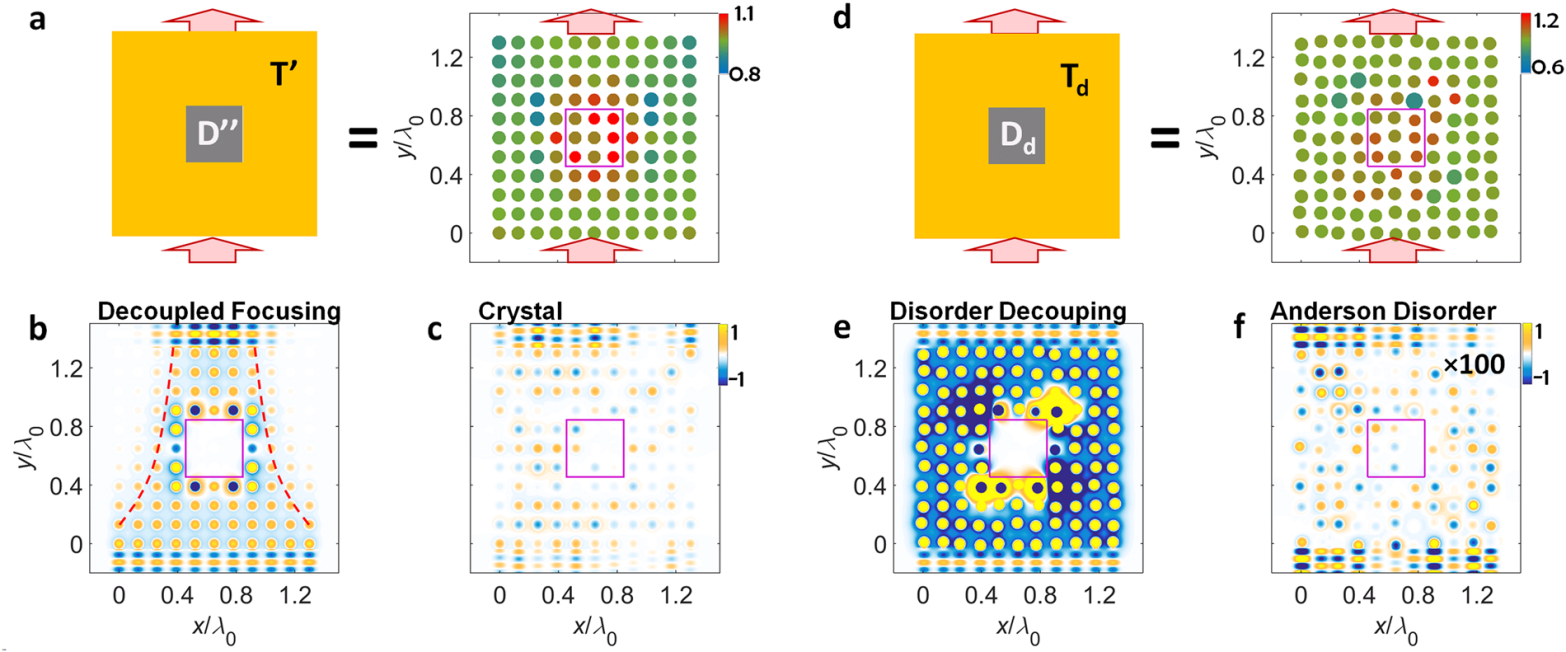
Demonstration of eigenstate decoupling with functionalities of focusing and disorder-resistant transport. (**a–c**) The decoupling with wave focusing (T’): (**a**) a schematic, (**b**) the field profile in the eigenstate decoupling system, and (**c**) the field profile in the ordinary crystal system. (**d–f**) The decoupling in the disordered system (D_d_, T_d_): (**d**) a schematic, (**e**) the field profile in the eigenstate decoupling system, and (**f**) the field profile in ordinary Anderson off-diagonal disorder system. The position of each resonator in (**d–f**) is randomly deformed for *x* and *y* axes, with the ±*Λ*_0_/10 maximum deformation for the original periodicity *Λ*_0_. The field amplitude in (**f**) is magnified (×100) for the presentation. All other parameters are the same as those in Fig. 2.

Based on the design methodology in Supplementary Note 1, we demonstrate the decoupling in coupled optical systems (Figs 2 and 3). Without loss of generality, we employ the system of coupled titanium oxide (TiO_2_) circular resonators embedded in an indium antimonide (InSb) crystalline compound, operating in the terahertz regime with transverse magnetic (TM) monopole resonances. We control the radii of resonators and their locations to adjust the resonant frequency *f* and coupling *κ*, respectively (see the detailed design in Supplementary Note 2). We investigate the 11 × 11 coupled resonator square lattice, encompassing the 3 × 3 decoupled region at the center of the system (the ‘decoupled’ region D in Fig. 2. Its surrounding ‘transport’ region is denoted as T). The binary random self-energy is applied to the resonators in the region D for clarity; the elements inside the decoupled region have one of the two self-energy values (or resonant frequencies) *f* = *f*_0_ or *f* = 1.1·*f*_0_ with the same probability (*f*_0_: operating frequency). By following the methodology in Supplementary Note 1, the self-energy distribution of the region T is derived both for the decoupling of the region D, and for the designed spatial profile of wave transport which determines the shapes of input and output waves. To demonstrate the decoupling operation, we compare the results from the eigenstate decoupling environments (Figs 2b,e and 3b,e) with those from the ordinary crystal environments which have identical elements at the region T (Figs 2c,f and 3c,f).

Figure 2 shows the cases of planewave spatial profiles, demonstrating the decoupling wave transfer for the different sets of elements inside the target region D. In general, the detailed configuration of the self-energy distribution strongly affects the wave transport in a coupled optical system, because the self-energy determines not only the phase evolution inside each element but also the coupling efficiency between elements^20^. However, regardless of the configuration of the target region D (D ≠ D’ in Fig. 2a,d), the eigenstate decoupling systems provide the perfect planewave transfer (Fig. 2b,e) with the same transport region T configuration, in sharp contrast to strong scattering and spatial incoherence in the crystal platforms the light flow of which has also strong dependence on the configuration of the region D (D ≠ D’ in Fig. 2c,f). This result demonstrates that the decoupling eigenstate designed by the methodology in Supplementary Note 1 successfully neglects the self-energy perturbation inside the target region, realizing the “target decoupling” based on the form of the eigenstate. In Supplementary Notes 3 and 4, we also investigate the stable operation regime of the proposed target decoupling, by analyzing the tolerance with respect to the perturbation in incident waveforms (Supplementary Note 3) and the fabrication errors exerted on the resonant frequency *f* and coupling *κ* which are determined by the radius of each resonator and the distance between resonators, respectively (Supplementary Note 4).

### Target decoupling with functionalities

As shown in the closed form of Eq. (S5) in Supplementary Note 1, the self-energy distribution is uniquely defined for ‘any’ nodeless eigenstate which satisfies the decoupling condition (*ψ* = 0) in the region D. Conversely, by controlling the self-energy of the environmental region T (T’ in Fig. 3a), the molding of the spatial form of wave flows becomes possible while preserving the scattering-free condition around the region D; as shown in the wave focusing example in Fig. 3b (compared to the random scattering in the ordinary environment of Fig. 3c). We thus note that designer wave flows with optical functionalities, such as focusing, beam splitting, and mode conversion, can be achieved, regardless of the perturbation inside the target decoupled region D.

The main strength of the eigenstate decoupling is the high applicability to ‘any’ coupling networks which may not have the spatial symmetry, in contrast to the indispensable spatial symmetry in the Dirac point cloaking^13^ or parity-time-symmetric invisibility^14–16^. The evidence is shown in Fig. 3d–f, demonstrating the decoupling in the system which has the off-diagonal disorder^22, 23^ from the random deformation of each resonator position (disordered coupling both in D_d_ and T_d_ regions in Fig. 3d). Perfect coherent transmission (Fig. 3e) is achieved as same as the cases in the lattice structure, overcoming the incoherent blockade of wave transport from Anderson localization (35 dB enhancement from 0.03% transmission at Fig. 3f). Distinct from previous cloaking methods in crystals^13–16^ which necessitate the strict spatial symmetry for the position of each optical element, the eigenstate decoupling method allows for the decoupling inside randomly distributed resonator systems, surprisingly, compensating the Anderson blockade from the off-diagonal disorder, as an example of the designer disorder^24–28^. We note that in spite of the requirement of the designed self-energy distribution, the decoupling without the spatial symmetry provides a novel route to ‘hiding’ elements in coupled systems.

### Statistical analysis of target decoupling

**Figure 4:**
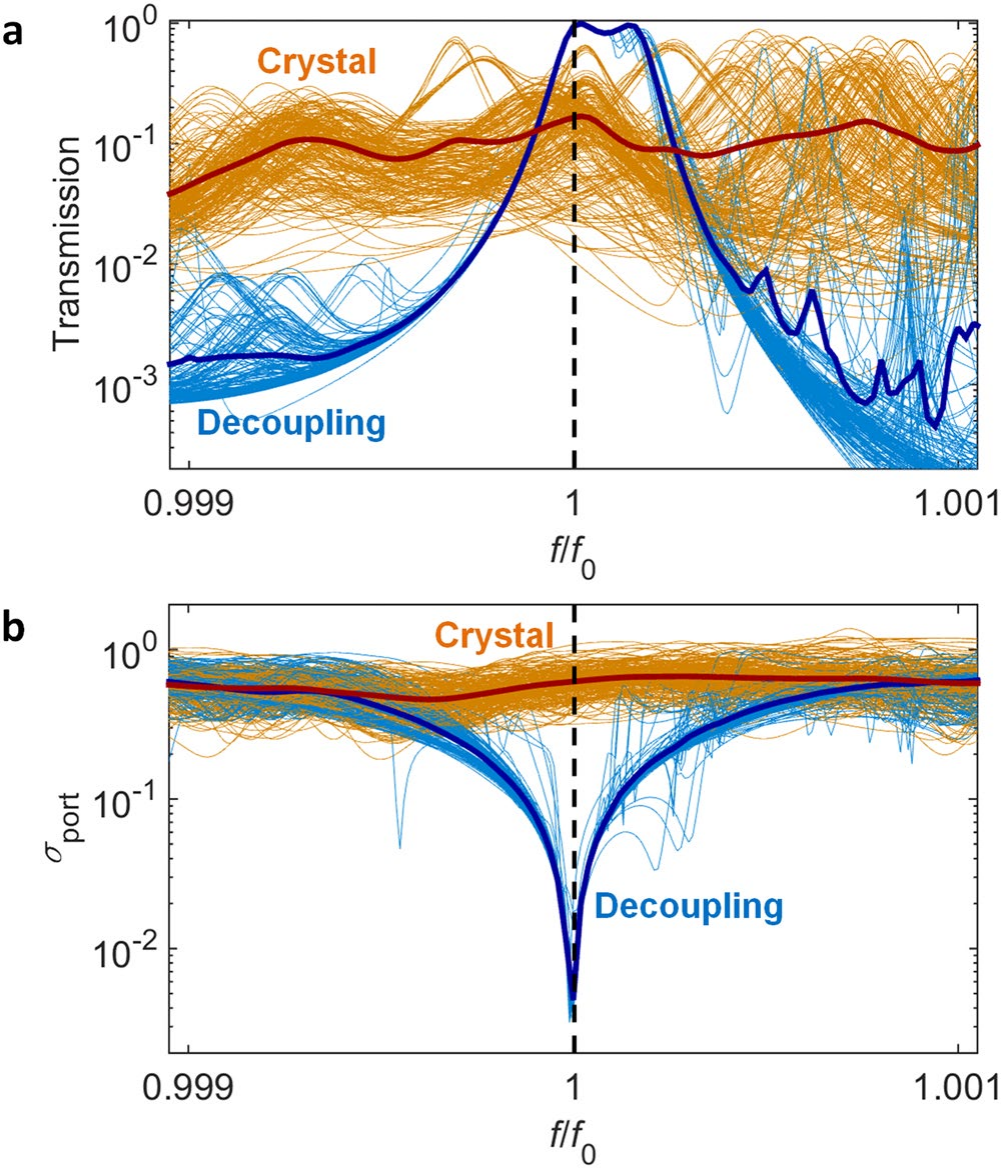
Statistical spectral analysis of eigenstate decoupling. (**a**) Transmission and (**b**) amplitude fluctuation spectra for the decoupling system (light blue thin lines) and the ordinary crystal system (orange thin lines), for the ensemble of 2^9^ samples. The fluctuation *σ*_port_ in (**b**) is the standard deviation of output field amplitude for 11 ports, normalized by the averaged amplitude (*σ*_port_ = 0 for ideal planewave). Blue and red thick lines in (**a,b**) denote the averaged results for 2^9^ samples of each system. Black dashed line depicts the design frequency.

To illustrate the stability and spectral property of the eigenstate decoupling method applied in Fig. 2, the statistical spectral analysis of the decoupling system is shown in Fig. 4. For 9 decoupled elements (region D in Fig. 2a,d) which have binary random resonant frequencies of *f* = *f*_0_ and *f* = 1.1·*f*_0_, the statistical ensemble of 2^9^ samples having the identical region T in Fig. 2a,d is realized to examine the coherence and transmission over the decoupling system (each thin lines in Fig. 4a,b). The spatial profile of the transmitted wave is quantified by measuring the standard deviation of output field amplitude *σ*_port_ for output ports (*σ*_port_ = 0 for ideal planewave). We note that about 94% of average transmission (Fig. 4a, blue thick line) with the almost uniform spatial profile (Fig. 4b) is achieved near the operating frequency, robust to the random alteration of the decoupled region (~0.040% standard deviation for the transmission): in sharp contrast to the performance of the ordinary crystal system (~16% transmission with 11% standard deviation).

**Figure 5:**
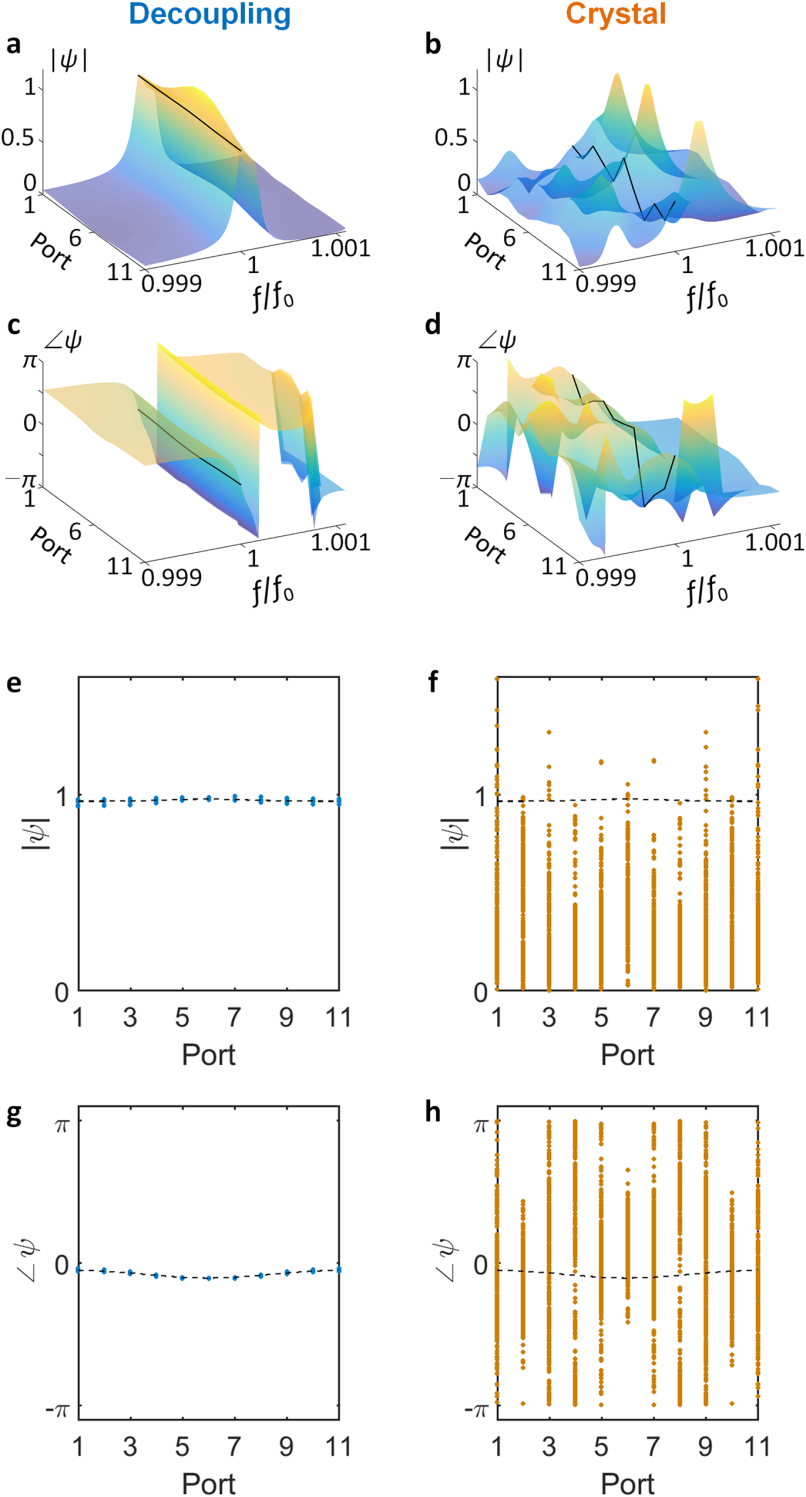
Spatial coherence of output flows through eigenstate cloaking. The amplitude (**a,b**) and phase (**c,d**) of the output field is plotted as a function of frequency and output positions, for an example of decoupling (**a,c**) and ordinary systems (**b,d**). Black lines denote the results at the operating frequency *f*_0_. (**e,f**) The amplitudes and (**g,h**) phases of the output field at each output port, for the (**e,g**) eigenstate decoupling and (**f,h**) ordinary crystal systems (at operating frequency *f*_0_). Each dot denotes a sample of a statistical ensemble (2^9^ samples), and black dashed lines represent the averaged results of 2^9^ samples.

The output flow through the decoupling system preserves excellent spatial coherence as well (Fig. 5). Compared to incoherent scattering with random phase and amplitude in the ordinary crystal system (Fig. 5b,d), the decoupling system of Fig. 2a,d derives the unity amplitude (Fig. 5a) and constant phase (Fig. 5c) at the output, independent from the random alteration of the decoupled region. Figure 5e–h also demonstrates the spatial coherence of the output field at the operating frequency, for the statistical ensemble of 2^9^ samples, by comparing the cases of the eigenstate decoupling system (Fig. 5e,g) and the ordinary crystal system (Fig. 5f,h). As shown in almost flat amplitude and phase distributions in the eigenstate decoupling system, the proposed system preserves all of the spatial information of the incident wave regardless of the detailed composition of the decoupled region, realizing the complete decoupling condition.

## Discussion

In summary, we proposed a new class of decoupling techniques for photonic integrated circuits, the macroscopic ‘decoupling’ of optical elements, by exploiting the system eigenstate with destructive interference regions. Based on the statistical analysis, we proved that the eigenstate decoupling method stably hides optical elements inside the coupled system, simultaneously allowing coherent wave transport with desired spatial profiles. Distinct from previous achievements in symmetry-based cloaking^13–16^, we also demonstrated the decoupling in disordered systems with the suppressed Anderson localization, as an example of the designer disorder^24–31^. Although we demonstrated the target decoupling in the THz platform utilizing subwavelength TiO_2_ resonators, the concept can be directly extended to visible or infrared regimes, when unit optical elements of the system satisfy the weak coupling condition^20^.

The eigenstate decoupling method provides excellent flexibility to the waveform molding in coupled optical systems, with the control of transport region elements. Likewise the global scattering increase in spectral domain as observed in most of cloaking structures^32^ (except few extreme cases such as diamagnetic and superconducting cloaks^32^), the bandwidth problem in our system is the engineering subject which can be improved by alleviating the strict decoupling condition. Our approach, separating target elements from the other region in coupling networks using moderate material/structural parameters, also possesses the link with the selective target control^33–35^ in network theory. From the deterministic operation based on the designer eigenstate, the applications exploiting multimodal^36, 37^ or continuous^38–40^ non-Hermitian potentials can also be envisaged for the defect-resistant realization of lasers or absorbers.

## Electronic supplementary material

Supplemental Material for ‘Target decoupling in coupled systems resistant to random perturbation’
