# Supplementary material for: Target decoupling in coupled systems resistant to random perturbation
Source: Sci Rep. 2017 May 19;7:2139. doi: 10.1038/s41598-017-01241-1 (PMC5438401; doi:10.1038/s41598-017-01241-1)
Supplement: Supplementary file 1 — Supplemental Material for ‘Target decoupling in coupled systems resistant to random perturbation’ [file 41598_2017_1241_MOESM1_ESM.pdf]

Target decoupling in coupled systems resistant to random perturbation

Sunkyu Yu, Xianji Piao, and Namkyoo Park*

Photonic Systems Laboratory, Department of Electrical and Computer Engineering, Seoul National University, Seoul 08826, Korea

**E-mail address for correspondence: nkpark@snu.ac.kr*

Supplemental Material

Supplementary Note 1. Design of an *N*-atomic coupled system for a decoupling eigenstate

Supplementary Note 2. Design of transverse magnetic monopole resonances

Supplementary Note 3. Effect of perturbed incidences

Supplementary Note 4. Effect of fabrication errors

Supplementary Note 1. Design of an *N*-atomic coupled system for a decoupling eigenstate

Extending the discussion in the main text, here we show the design procedure (Fig. S1) of the *N*-atomic system which possesses a decoupling eigenstate. The Hamiltonian equation for the *N*-atomic system composed of weakly coupled elements is[1-4]

$$\begin{bmatrix} \rho_1 & \kappa_{12} & \cdots & \kappa_{1N} \\ \kappa_{21} & \rho_2 & \cdots & \kappa_{2N} \\ \vdots & \vdots & \ddots & \vdots \\ \kappa_{N1} & \kappa_{N2} & \cdots & \rho_N \end{bmatrix} \begin{bmatrix} \psi_1 \\ \psi_2 \\ \vdots \\ \psi_N \end{bmatrix} = \rho \cdot \begin{bmatrix} \psi_1 \\ \psi_2 \\ \vdots \\ \psi_N \end{bmatrix}. \quad \text{(S1)}$$

For the given coupling network (κ_{jk}, Fig. S1a), we will derive the necessary form of the decoupling eigenstate $\Psi = [\psi_1, \psi_2, \ldots, \psi_N]^T$, to determine the corresponding self-energy[4] $\Omega = [\rho_1, \rho_2, \ldots, \rho_N]^T$.

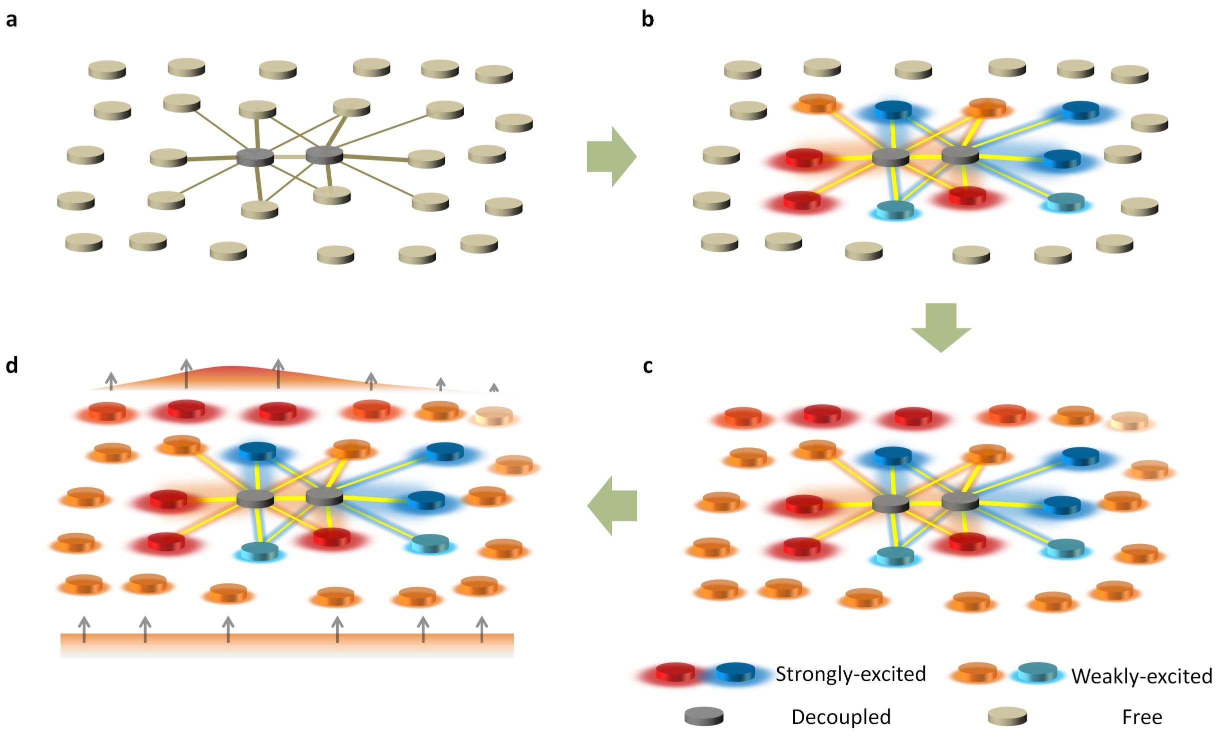

Figure S1. The design procedure of an *N*-atomic system for a decoupling eigenstate. (**a**) The selection of target decoupled elements in the system which has the determined coupling network. The design of the field distribution in (**b**) nearby elements of target elements and (**c**) the rest tunable elements. (**d**) The wave flow through the system, realizing the scattering-free manipulation of the waveform. Couplings only around the decoupled elements are presented for clarity.

With *m* number of decoupled elements ($m \leq N$, $\psi = 0$) the indices of which constitute the set A, the set of nearby elements ($\psi \neq 0$) for each decoupled element can be defined as B_j ($j \in A$, e.g. A =

{5} and B_5 = {1,2,3,4,6,7,8,9} in Fig. S2a, for m = 1 and N = 9). Because at most nearest-neighbor and next-nearest-neighbor coupling coefficients are significant in realistic structures[5] (Fig. S1a) due to the exponential decay of evanescent coupling in space, each row of Eq. (S1) for decoupled elements derives the following condition of the destructive coupling interference as

$$\sum_{k \in B_j} \kappa_{jk} \cdot \psi_k = 0, \tag{S2}$$

where $j \in$ A, and the condition of $k \in B_j$ represents the nearby coupling ($\kappa_{jk} \sim 0$ for far-off elements of $k \notin B_j$, for the j-th decoupled element). Equation (S2) governs the necessary condition of the field amplitude in nearby elements (Fig. S1b), providing each equation for $j \in$ A which has the number of variables equal to the number of elements in B_j. Note that the degree of freedom (DOF) of Eq. (S2), the difference between the number of variables (field amplitude in nearby elements) and the number of constraints by equations (zero-field condition in decoupling elements), is determined by the number of nearby elements for each decoupled element (e.g. in Fig. S2b, two equations from m = 2, and 10 variables from nearby elements (light gray), lead to 8 DOF).

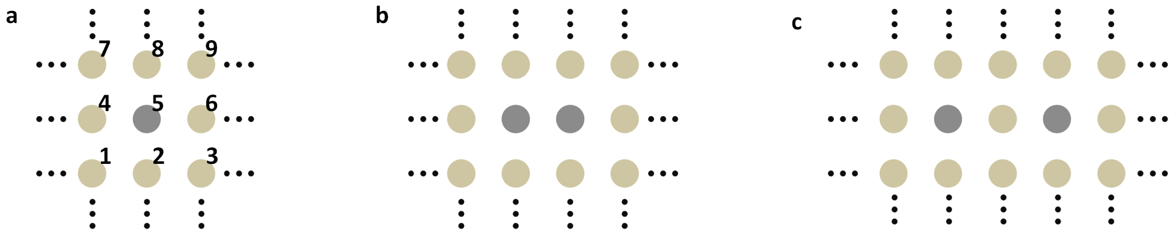

Figure S2. The schematics of decoupled (dark gray) and nearby (light gray) elements: (**a**) 7 DOF, (**b**) 8 DOF, and (**c**) 11 DOF.

After the nearby elements for all of the decoupled elements are determined to satisfy Eq. (S2) (Fig. S1b, the elements in B_j for all $j \in$ A), the other region of the decoupling eigenstate can then be designed (Fig. S1c). Except the rows of decoupled indices for Eq. (S2), the other part of Eq. (S1) has the form of

$$\begin{bmatrix} \rho_{s\text{-}1} & \kappa_{s\text{-}1,s\text{-}2} & \cdots & \kappa_{s\text{-}1,s\text{-}(N-m)} \\ \kappa_{s\text{-}2,s\text{-}1} & \rho_{s\text{-}2} & \cdots & \kappa_{s\text{-}2,s\text{-}(N-m)} \\ \vdots & \vdots & \ddots & \vdots \\ \kappa_{s\text{-}(N-m),s\text{-}1} & \kappa_{s\text{-}(N-m),s\text{-}2} & \cdots & \rho_{s\text{-}(N-m)} \end{bmatrix} \begin{bmatrix} \psi_{s\text{-}1} \\ \psi_{s\text{-}2} \\ \vdots \\ \psi_{s\text{-}(N-m)} \end{bmatrix} = \rho \cdot \begin{bmatrix} \psi_{s\text{-}1} \\ \psi_{s\text{-}2} \\ \vdots \\ \psi_{s\text{-}(N-m)} \end{bmatrix}, \tag{S3}$$

where the new index (s-p) $\notin$ A and $1 \leq$ (s-p) $\leq N$. For the subset of the decoupling eigenstate Ψ_s = [ψ_{s-1}, ψ_{s-2},…, $\psi_{s-(N-m)}$]^T, although the nearby elements of decoupled elements ((s-p) $\in$ B_j for all $j \in$ A, red and blue elements in Fig. S1b) are already determined for the decoupling (Eq. (S2)), the other elements ((s-p) $\notin$ B_j for any $j \in$ A, light gray elements in Fig. S1c) can be freely set to achieve desired optical functionalities (e.g. steered beam focusing in Fig. S1d), finally defining Ψ_s and then Ψ where $\psi_j = 0$ for $j \in$ A.

From the decoupling eigenstate Ψ with the desired functionality, we can then achieve the corresponding self-energy distribution Ω = [ρ_1, ρ_2,…, ρ_N]^T. The $(N-m) \times (N-m)$ matrix equation of Eq. (S3), off-diagonal terms of which have given values, can be recast into the form of

$$\begin{bmatrix} \psi_{s-1} & 0 & \cdots & 0 \\ 0 & \psi_{s-2} & \cdots & 0 \\ \vdots & \vdots & \ddots & \vdots \\ 0 & 0 & \cdots & \psi_{s-(N-m)} \end{bmatrix} \begin{bmatrix} \rho_{s-1} \\ \rho_{s-2} \\ \vdots \\ \rho_{s-(N-m)} \end{bmatrix} = \left(\rho \cdot I - \begin{bmatrix} 0 & \kappa_{s-1,s-2} & \cdots & \kappa_{s-1,s-(N-m)} \\ \kappa_{s-2,s-1} & 0 & \cdots & \kappa_{s-2,s-(N-m)} \\ \vdots & \vdots & \ddots & \vdots \\ \kappa_{s-(N-m),s-1} & \kappa_{s-(N-m),s-2} & \cdots & 0 \end{bmatrix} \right) \begin{bmatrix} \psi_{s-1} \\ \psi_{s-2} \\ \vdots \\ \psi_{s-(N-m)} \end{bmatrix}, \quad \text{(S4)}$$

where I is the $(N-m) \times (N-m)$ identity matrix. Because the diagonal matrix $diag(\Psi_s)$ has its inverse due to $\psi_{(s-p)} \neq 0$ for all (s-p) $\notin$ A, Eq. (S4) derives the required self-energy distribution Ω_s = [ρ_{s-1}, ρ_{s-2},…, $\rho_{s-(N-m)}$]^T except the decoupled elements as,

$$\begin{bmatrix} \rho_{s-1} \\ \rho_{s-2} \\ \vdots \\ \rho_{s-(N-m)} \end{bmatrix} = \begin{bmatrix} \psi_{s-1} & 0 & \cdots & 0 \\ 0 & \psi_{s-2} & \cdots & 0 \\ \vdots & \vdots & \ddots & \vdots \\ 0 & 0 & \cdots & \psi_{s-(N-m)} \end{bmatrix}^{-1} \left(\rho \cdot I - \begin{bmatrix} 0 & \kappa_{s-1,s-2} & \cdots & \kappa_{s-1,s-(N-m)} \\ \kappa_{s-2,s-1} & 0 & \cdots & \kappa_{s-2,s-(N-m)} \\ \vdots & \vdots & \ddots & \vdots \\ \kappa_{s-(N-m),s-1} & \kappa_{s-(N-m),s-2} & \cdots & 0 \end{bmatrix} \right) \begin{bmatrix} \psi_{s-1} \\ \psi_{s-2} \\ \vdots \\ \psi_{s-(N-m)} \end{bmatrix}. \quad \text{(S5)}$$

Because the satisfaction of Eq. (S2) and Eq. (S3) corresponds to the satisfaction of Eq. (S1), the self-energy distribution Ω which has the subset of Ω_s from Eq. (S5) and ‘arbitrary’ values for the Ω_s’s complementary set, derives the decoupling eigenstate Ψ which has $\psi_j = 0$ for $j \in$ A and Ψ_s for the other part. We note that the eigenstate Ψ in the potential Ω therefore achieves the decoupling (scattering-free for arbitrary ρ_j of $j \in$ A) and the functionality (designed Ψ_s, Fig. S1d) at the same time.

Supplementary Note 2. Design of transverse magnetic monopole resonances

In the main text, we utilize the transverse magnetic 'monopole' mode (H_z and $E_{r,\varphi}$ fields with $\partial H_z / \partial\varphi = 0$) of two-dimensional circular resonators (the refractive index $n = n_1$ for dielectric core and $n = -in_2$ for metallic background), which derives the coupling coefficient dependent only on the distance between resonators. From the governing wave equation

$$r \cdot \frac{\partial^2 H_z}{\partial r^2} + \frac{\partial H_z}{\partial r} + k_0{}^2 n^2 r \cdot H_z = 0, \tag{S6}$$

where $k_0 = 2\pi \cdot f \,/\, c$ is the free-space wavenumber, we seek the localized solution of H_z without singularity, which has the form of

$$\begin{aligned} H_z &= c_1 \cdot J_0(n_1 k_0 r) \qquad (\text{for } r \le r_0) \\ &= c_2 \cdot K_0(n_2 k_0 r) \qquad (\text{for } r > r_0) \end{aligned}, \tag{S7}$$

where J_0 and K_0 each denotes the zeroth-order Bessel and modified Bessel function. Equation (S7) then derives the resonance condition of

$$n_1 \cdot J_0(n_1 k_0 r_0) \cdot K_1(n_2 k_0 r_0) + n_2 \cdot J_1(n_1 k_0 r_0) \cdot K_0(n_2 k_0 r_0) = 0, \tag{S8}$$

from the electromagnetic boundary condition ($H_{z1} = H_{z2}$ and $E_{\varphi 1} = E_{\varphi 2}$).

We assume a titanium oxide[6,7] core (TiO_2, refractive index $n = 10$) and an indium antimonide crystalline compound[8,9] background (InSb, $n = 0.3619 - 5.107i$), for the operation in the terahertz regime (near 1.1THz). Equation (S8) then has a solution for $k_0 \cdot r_0 = 0.275$, which derives the necessary core radius for the resonant frequency f_0 as

$$r_0 = 0.275 \cdot \frac{c}{2\pi f_0}. \tag{S9}$$

For example, the TiO_2 core radius is 11.6μm for the resonant frequency $f_0 = 1.13$THz.

Same as the previous work[4], coupling coefficients between resonators are calculated by COMSOL Multiphysics, deriving the exponential relation $\kappa/f_0 \sim 0.959 \cdot exp(-36 \cdot d/\lambda_0)$ for an excellent fit to κ in the weak-coupling regime (here, $\kappa/f_0 < 1/40$, d: distance between resonators).

Supplementary Note 3. Effect of perturbed incidences

To examine the stability of the eigenstate decoupling method, we analyze the operation in Fig. 2 in the main text for perturbed wave incidences. As an example, we define the incidence as $\Psi_{inc} = [1, 1, \ldots, 1]^T + \Delta_{inc} \cdot [\psi_{inc\text{-}1}, \psi_{inc\text{-}2}, \ldots, \psi_{inc\text{-}11}]^T$ where $\psi_{inc\text{-}k} = u[-1,1]$ ($k = 1, 2, \ldots, 11$) is determined by the uniform probability density function u, and Δ_{inc} is the magnitude of the perturbation ($\Delta_{inc} = 0$ for the results of Fig. 2 in the main text). The representative spatial profiles of input incidences for $\Delta_{inc} = 0$, 1, and 2 are shown in Fig. S3a-c, respectively.

The result is shown in Fig. S3d, presenting the transmission with respect to the magnitude of the perturbation Δ_{inc}. As seen, although the transmission through the decoupling system decreases for increasing Δ_{inc}, the system still supports much higher transmission than that of the ordinary crystal system, due to the perfect transparency of the designed state.

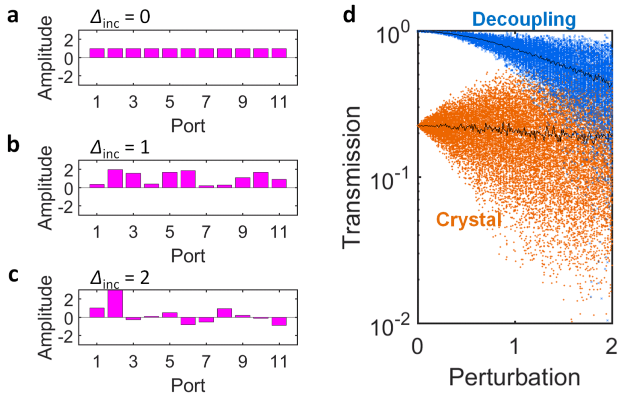

Figure S3. Effect of the perturbation on the incident wave. The profiles of input waves for (**a**) $\Delta_{inc} = 0$, (**b**) $\Delta_{inc} = 1$, and (**c**) $\Delta_{inc} = 2$. (**d**) Transmission of the decoupling system (blue) and ordinary crystal system (orange). Each dot denotes the transmission for an each random incidence. Black solid lines denote the ensemble average of 100 random samples.

Supplementary Note 4. Effect of fabrication errors

We now assume the fabrication errors in the distance between resonators and the radius of each resonator (Fig. S4a), to examine the stability of the eigenstate decoupling system in Fig. 2 in the main text. The distance is set to be $d_{err} = d\cdot(1 + \mathit{\Delta}_d\cdot u[-0.5,0.5])$ and the radius of each resonator is set to be $r_{0\text{-err}} = r_0\cdot(1 + \mathit{\Delta}_r\cdot u[-0.5,0.5])$ where d and r_0 are the original distance and radius values, and $\mathit{\Delta}_d$ and $\mathit{\Delta}_r$ are the magnitude of fabrication errors, respectively. The corresponding coupling coefficients and resonant frequencies are obtained by the analysis in Supplementary Note 2.

The result for each error is shown in Fig. S4b,c, presenting the transmission with respect to the magnitude of the fabrication errors $\mathit{\Delta}_d$ and $\mathit{\Delta}_r$, and also comparing with the results from ordinary crystal systems. As seen, although the eigenstate decoupling system is more resistant to the distance error (or coupling error until ~5%), the stable regime of the eigenstate decoupling system is fragile to the radius error (or self-energy error, < 0.7%). This phenomenon, the sensitivity to self-energy errors, is an inherent property of weakly-coupled systems[10], because the self-energy mismatch prohibits the complete energy transfer between optical elements.

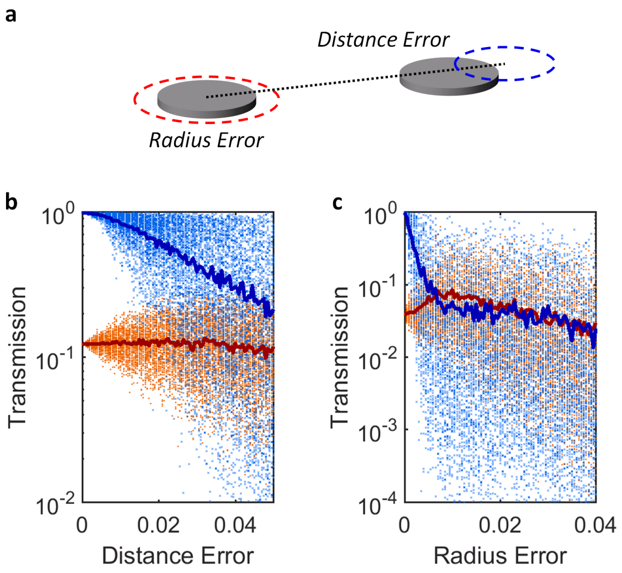

Figure S4. Effect of fabrication errors. (**a**) A schematic of the fabrication errors on the resonator radius and the distance between resonators. (**b,c**) Transmissions of the decoupling system (blue) and ordinary crystal system (orange) for the (**b**) distance error and (**c**) resonator radius error. Each dot denotes the transmission for an each case of fabrication errors. Solid lines denote the ensemble average of 100 random samples.

References

1. Christodoulides, D. N., Lederer, F. & Silberberg, Y. Discretizing light behaviour in linear and nonlinear waveguide lattices. *Nature* **424**, 817-823 (2003).

2. Longhi, S. Quantum-optical analogies using photonic structures. *Laser Photon. Rev.* **3**, 243-261 (2009).

3. Mrejen, M. *et al.* Adiabatic elimination-based coupling control in densely packed subwavelength waveguides. *Nat. Commun.* **6**, 7565, doi:10.1038/ncomms8565 (2015).

4. Yu, S., Piao, X., Hong, J. & Park, N. Metadisorder for designer light in random systems. *Sci. Adv.* **2**, e1501851, doi:10.1126/sciadv.1501851 (2016).

5. Keil, R. *et al.* Direct measurement of second-order coupling in a waveguide lattice. *arXiv preprint arXiv:1510.07900* (2015).

6. Matsumoto, N. *et al.* Analysis of dielectric response of TiO2 in terahertz frequency region by general harmonic oscillator model. *Jpn. J. Appl. Phys.* **47**, 7725 (2008).

7. Berdel, K., Rivas, J. G., Bolívar, P. H., De Maagt, P. & Kurz, H. Temperature dependence of the permittivity and loss tangent of high-permittivity materials at terahertz frequencies. *IEEE Trans. Microw. Theory Techn.* **53**, 1266-1271 (2005).

8. Howells, S. & Schlie, L. Transient terahertz reflection spectroscopy of undoped InSb from 0.1 to 1.1 THz. *Appl. Phys. Lett.* **69**, 550-552 (1996).

9. Isaac, T. H., Rivas, J. G., Sambles, J. R., Barnes, W. L. & Hendry, E. Surface plasmon mediated transmission of subwavelength slits at THz frequencies. *Phys. Rev. B* **77**, 113411 (2008).

10. Haus, H. A. *Waves and fields in optoelectronics*. (Prentice-Hall Englewood Cliffs, NJ, 1984).
